# Outlines of Lectures on Human Physiology

**Published:** 1818-02

**Authors:** 


*Outlines of Lectures on Human Physiology.* By John Gordon, M.D. Lecturer on Anatomy and Surgery, and on the Institutions of Medicine, Edinburgh. pp. 200. 8vo. Edinburgh, 1818.

When we saw this work announced, we sent to our Bookseller for a copy, in all the precipitation of eagerness — ready to hail it as a worthy contribution to English Physiology, and as a good set-off against the "Precis Elementaire" of a rival nation. But our readers will understand the nature of our disappointment, when we tell them, in significant though vulgar language, that we found ourselves *wofully bit!*

We have a no less tender care for the earthly, than for the intellectual treasures of our fellow-lieges, and would admonish all and every of them to keep a strict guard over their purse-strings, and to *look*, ere they pull out the six resplendent shillings and *buy!* They will do well to take warning from the hoax which our inconsiderateness has put upon us:

"Felix quem faciunt aliena pericula cautum."

In sober earnest, this volume, about which our expectations were so sanguine, (for we heard more than twelve months since of the author's intention to publish on Physiology,) — this volume, we say, so long expected, when it came at last, turned out nought but a skeleton—a frame-work (call it what you will),—or lengthened Syllabus — of a Course of Lectures on Physiology, delivered by the author at Edinburgh. Since we are so many leagues distant from that intellectual capital, and consequently have it not in our power to seat ourselves "at the feet of Gamaliel," we must e'en be content to "burst in ignorance" touching the various matters which this book advertises. The book, nevertheless, is a good book, and fair to the eye. It is but justice to say, that its contents are, upon the whole, very skilfully arranged, and seem to be well got up "ad captandum."

Henceforth let no man say unto himself that miracles have ceased. Now-a-days an advertisement can dilate itself to the extraordinary magnitude of a goodly octavo of two hundred pages! 'Tis strange; but what then? "Non aliter fit, avite, liber." Would to Heaven, that the order of this curious transmutation were now and then reversed! that some northern publications, of recent date, had shrunk, while in their nascent state, and been condensed, from a swaggering volume, to the more *just* and more humble limits of an advertisement. Had this been the case, we seriously think that medical science would have profited not a whit the less. Perhaps, too, the author's fame would have been better consulted by it: but that is his affair, not ours; therefore, it is for him to look to't.

